# *Typhonium boonkerdii* (Araceae), a Distinctive New Geophytic Species from Northeastern Thailand

**DOI:** 10.3390/life16101621

**Published:** 2026-09-26

**Authors:** Wilawan Promprom, Phukphon Munglue, Wannachai Chatan

**Affiliations:** 1 Department of Biology, Faculty of Science, Mahasarakham University, Kantharawichai District, Maha Sarakham 44150, Thailand; wilawan.pp@msu.ac.th; 2 Plant and Innovation Research Unit, Mahasarakham University, Kantharawichai District, Maha Sarakham 44150, Thailand; phukphon.m@ubru.ac.th; 3 Program of Biology, Faculty of Science, Ubon Ratchathani Rajabhat University, Ubon Ratchathani 34000, Thailand

**Keywords:** Araceae, aroid, conservation, geophyte, Indochina, Mukdahan, new species, nomenclature, plant diversity, taxonomy

## Abstract

Botanical exploration of northeastern Thailand has revealed substantial undocumented diversity within *Typhonium* (Araceae). Here, *T. boonkerdii* Chatan & Promprom is described and illustrated as a new geophytic species from Mukdahan Province. The taxonomic treatment is based on study of living plants during vegetative, flowering, and fruiting stages, together with comparative examination of herbarium specimens and relevant taxonomic literature. *Typhonium boonkerdii* is morphologically most similar to *T. cucullatum* but is distinguished by its shortly acute to shortly acuminate leaf-blade apex, longer and broader pistillate zone comprising 4–5 rows of densely congested pistils, larger ovaries and stigmas, broader sterile interstice, and mutually distant staminodes with downward- to slightly downward-directed apices. The new species further differs in having a longer staminate zone with 6–7 whorls of stamens, an appendix with an abruptly but slightly expanded base, and a longer stipe. Although both species show a similar sequence of spathe-limb movement during anthesis, the consistent combination of characters clearly supports the recognition of *T. boonkerdii* as a distinct species. The species is currently known from a single population in mixed deciduous forest at 300–400 m elevation. As the total population is estimated to comprise fewer than 50 mature individuals, the species is provisionally assessed as Critically Endangered (CR) following the IUCN Red List Categories and Criteria. A detailed description, photographic illustrations, distribution map, morphological comparison, and identification key are provided.

## 1. Introduction

*Typhonium* is a genus in the aroid family Araceae, comprising an estimated 70–100 species [1,2,3]. According to the current taxonomic circumscription adopted by Plants of the World Online, *Typhonium* is native from Mongolia and South Asia through Southeast and East Asia, the Malay Archipelago, and New Guinea to northern and eastern Australia [3]. Within Araceae, *Typhonium* belongs to tribe Areae, a group composed predominantly of terrestrial, seasonally dormant, tuberous or geophytic herbs [1,4,5,6].

Thailand is particularly notable for its high species diversity and degree of endemism and is recognized as a major center of *Typhonium* diversity, with more than 30 species recorded in the Flora of Thailand [1], underscoring its importance in the diversification of the genus. The treatment in the Flora of Thailand, together with earlier and subsequent taxonomic studies, has documented numerous species, several of which are narrowly endemic or known from only a few localities. These discoveries indicate that the diversity and geographical distribution of *Typhonium* in northeastern Thailand remain incompletely documented.

Among these recently described taxa, *T. cucullatum* is particularly notable for its elliptic, narrowly elliptic, or elliptic-lanceolate leaf blades and its distinctive spathe movement during anthesis [7]. At the early stage of pistillate anthesis, the limb of spathe is erect, gaping, and expanded, exposing the staminate zone. It then reflexes and curls strongly backward during pistillate anthesis before uncurling and gradually returning to an erect position during the late stage of pistillate anthesis. Thereafter, the limb curves strongly forward, forming a hood-like structure. This forward curvature is most pronounced during staminate anthesis and persists through post-staminate anthesis as the limb begins to wither [7]. The documentation of these sequential changes demonstrates that observations of living plants throughout anthesis can provide taxonomically informative characters that may not be adequately preserved in dried herbarium material.

The authors have conducted extensive botanical surveys in eastern and northeastern Thailand over many years, resulting in the discovery and documentation of numerous noteworthy Araceae taxa, with recent examples including *Colocasia sookchaloemiae* Chatan & Promprom, *Pycnospatha phanomdongrakensis* Chatan & Promprom and *T. mukdahanense* Chatan & Promprom [8,9,10]. Collectively, these findings demonstrate that continued fieldwork in these areas can substantially improve knowledge of the regional aroid flora. During fieldwork in Mukdahan Province, northeastern Thailand, the authors encountered a notable population of *Typhonium* in mixed deciduous forest. The Mukdahan plants are morphologically most similar to *T. cucullatum*, a species occurring in dry deciduous forest, with which they share a small, seasonally dormant geophytic habit, predominantly elliptic to narrowly elliptic leaf blades, a spathe clearly differentiated into a convolute basal portion and an elongate limb, a sterile interstice bearing free subulate staminodes, and a stipitate, narrowly elongate appendix [7]. However, the Mukdahan population differs consistently in several reproductive characters, particularly its longer and broader pistillate zone with more rows of pistils, larger ovaries and stigmas, broader sterile interstice, mutually distant staminodes with downward-curved to slightly downward-curved distal portions, longer staminate zone with more whorls of stamens, an appendix with a more or less abruptly expanded base, and a longer appendix stipe. The simultaneous occurrence of these stable differences supports recognition of the population in Mukdahan Province as a distinct species.

This study provides the formal taxonomic description and illustration of a new species of *Typhonium* from the northeastern part of Thailand. Details of its distinguishing morphological characters, geographic distribution, habitat, phenology, and provisional conservation assessment are provided, along with a detailed comparison with the morphologically most similar *T. cucullatum* and an identification key to related species.

## 2. Materials and Methods

Fieldworks were carried out in Mukdahan Province, northeastern Thailand, and adjacent areas between 2020 and 2025. A total of 36 field surveys were conducted, with six surveys undertaken each year. Approximately 40 individuals were observed in the known population. Plants were examined in their natural habitat during the vegetative, flowering, and fruiting periods whenever suitable material was available. Healthy, spatially separated individuals representing the observed range of morphological variation were selected for detailed examination and specimen preparation following standard taxonomic procedures. Fresh material was photographed and measured before preservation, and reproductive structures were preserved when necessary to facilitate detailed study. Voucher specimens were deposited in the BCU herbarium. Herbarium acronyms follow Index Herbariorum [11].

The morphological characters were examined using living plants and herbarium specimens. Larger organs were measured using a ruler, whereas smaller organs were examined under a stereomicroscope (Olympus SZ51; Olympus Corporation, Tokyo, Japan) and measured with an ocular micrometer. Terminology for vegetative and reproductive morphological characters followed that used in the relevant taxonomic literature [1,12,13,14,15,16]. Terminology relating specifically to the spathe morphology follows the distinction between the spathe base and limb, with the basal convolute part separated from the expanded distal part by a constricted region. The organization of the spadix was described according to its pistillate zone, sterile interstice, staminate zone, and appendix, following established usage in current taxonomic study of *Typhonium* [10]. This methodological approach is consistent with that applied in the description of *T. cucullatum* [7].

Particular attention was given to characters considered taxonomically informative in *Typhonium*, including plant height, tuber morphology, number of leaves, petiole and sheath dimensions, leaf-blade shape and venation, spathe size, coloration and movement during anthesis, dimensions of the fertile and sterile zones of the spadix, stigma form and colour, number and arrangement of staminodes, appendix shape and insertion, and fruit morphology. Characters of the spathe and spadix were recorded from fresh material whenever possible because their position, colour, and proportions may be distorted during dying.

Herbarium specimens of *Typhonium* species from Thailand and neighboring countries were examined either directly in herbarium collections or through digital images available on JSTOR Global Plants [17] and the online databases of the respective institutions. Collections housed in the following herbaria were consulted: A, AUU, B, BCU, BK, BKF, BM, BRI, C, CAL, CNS, DNA, E, G, K, KATH, KUN, L, M, MEL, NSW, P, PE, PERTH, SING, UTB, and WAG. Relevant regional floras, taxonomic treatments, revisions, articles and illustrations were also examined [1,7,10,18,19,20,21]. Particular attention was given to comparisons with *T. cucullatum* K.Z.Hein, P.Saensouk & Saensouk, the morphologically most similar species known from Sakon Nakhon Province, northeastern Thailand [7]. Diagnostic characters of *T. cucullatum* were assessed from the original description and images of the holotype (*Saensouk et al. s.n*., BKF) [7]. These characters were then compared directly with those observed in specimens of the Mukdahan population.

Information on distribution, vegetation type, substrate, elevation, population size, and potential threats was recorded during field surveys. Flowering and fruiting periods were determined from field observations and available specimen records. Locality data were used to prepare a distribution map of the new species. The preliminary conservation assessment was done according to the IUCN Red List Categories and Criteria and the current guidelines for their application [22].

## 3. Results

### 3.1. Taxonomic Treatment

***Typhonium boonkerdii* Chatan & Promprom, *sp. nov.***

**Figure 1:**
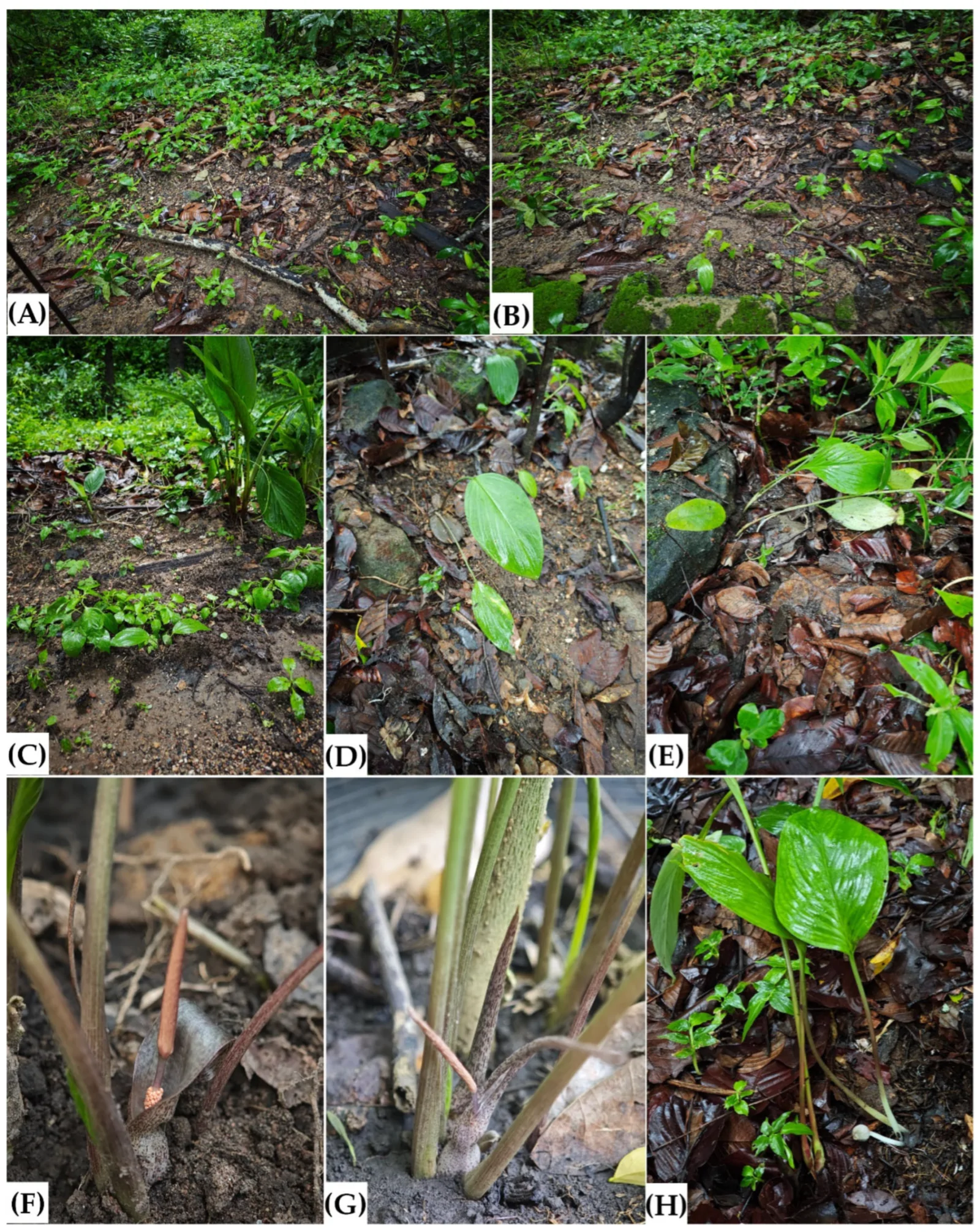
*Typhonium boonkerdii*. (**A**–**E**,**H**) Habitat and habit under natural field conditions; (**F**,**G**) habit of cultivated plants; (**G**) whole plant.

**Figure 2:**
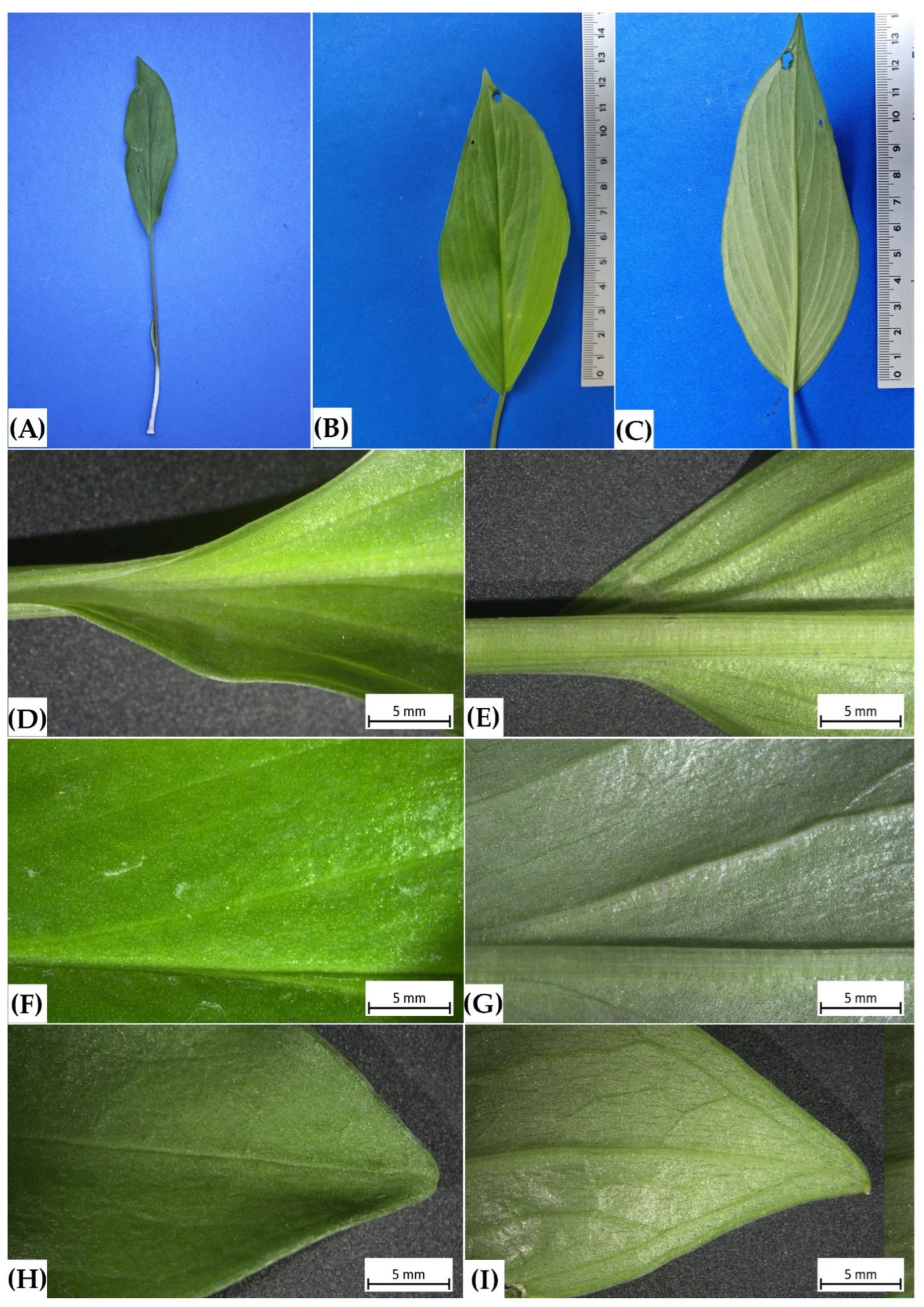
*Typhonium boonkerdii*. (**A**,**B**) Adaxial surface of leaf blades; (**C**) abaxial surface of leaf blade; (**D**,**E**) leaf-blade base, as viewed from the adaxial and abaxial surfaces, respectively; (**F**,**G**) leaf blade, as viewed from the adaxial and abaxial surfaces, respectively; (**H**,**I**) leaf blade apex, as viewed from the adaxial and abaxial surfaces, respectively.

**Figure 3:**
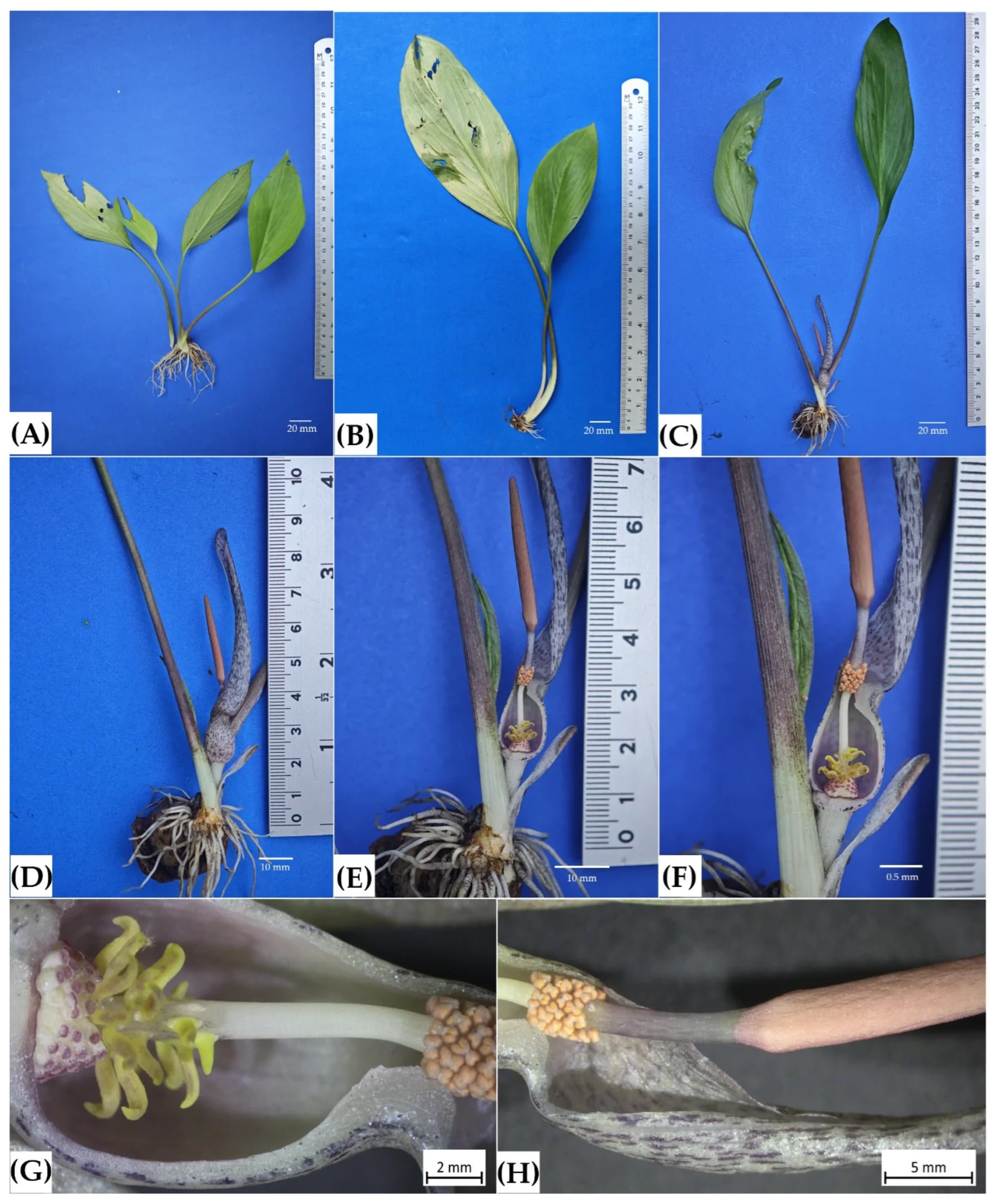
*Typhonium boonkerdii*. (**A**,**B**) Whole plants from different individuals; (**C**) whole plants with an inflorescence; (**D**–**F**) basal parts of plants bearing an inflorescence, with the spadix exposed in (**E**,**F**); (**G**) close-up of the basal part of the spadix.; (**H**) close-up of the upper part of the spadix.

**Figure 4:**
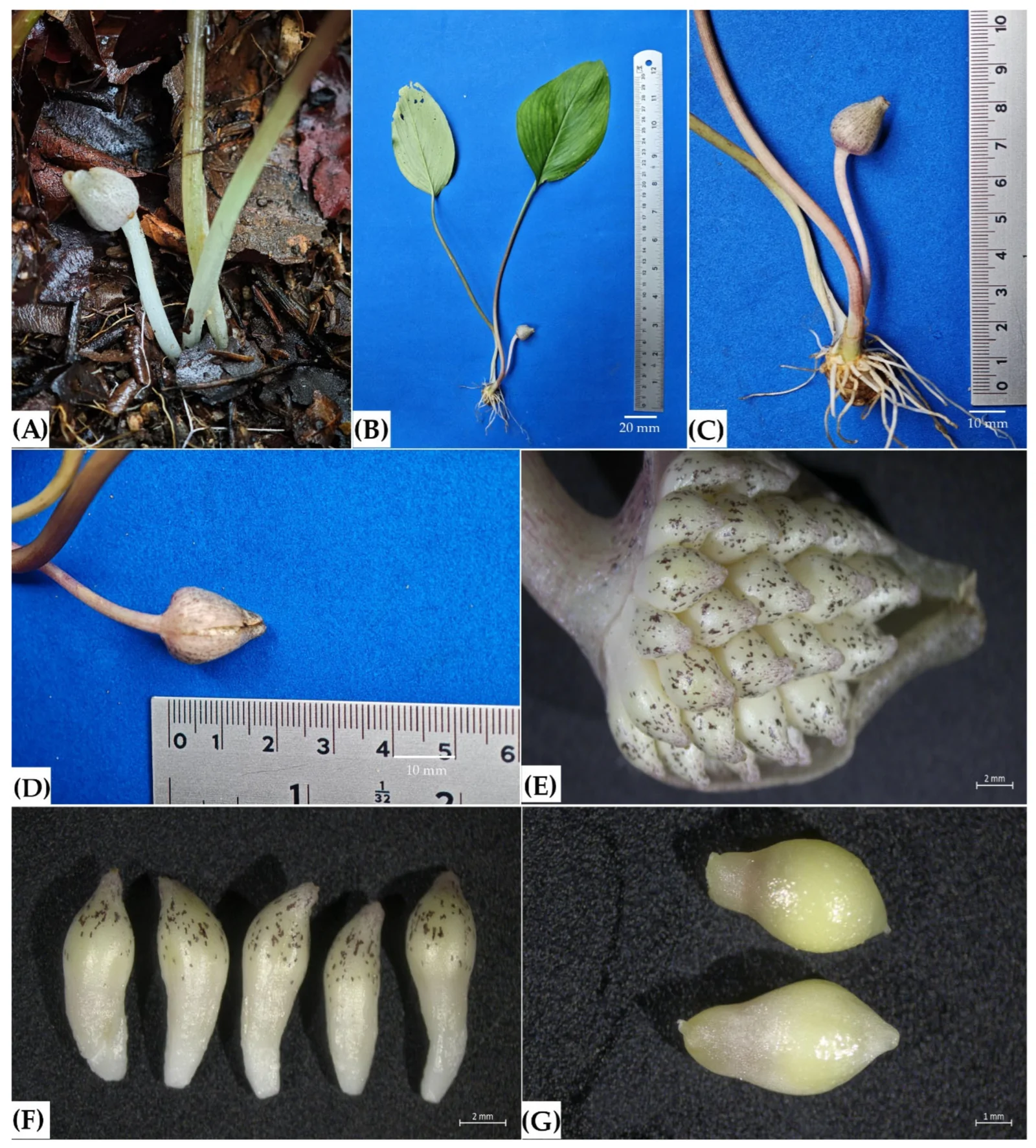
*Typhonium boonkerdii*. (**A**–**C**) Plants, each bearing a single inflorescence: (**A**) in the field; (**B**,**C**) in the laboratory; (**D**) infructescence enclosed by the lower portion of the spathe; (**E**) exposed infructescence; (**F**) berries; (**G**) seeds.

Figure 1, Figure 2, Figure 3 and Figure 4.

### 3.2. Type

Thailand. Northeastern: Mukdahan Province, 300–400 m elev., 16°47′54.9″ N 104°25′55.0″ E, June 2021, *W. Chatan 2810* (holotype BCU!; isotype BCU!)

### 3.3. Diagnosis

*Typhonium boonkerdii* most closely resembles *T. cucullatum* in overall morphology but differs in having short acute to shortly acuminate leaf-blade apices (vs. tapering into a long, slender acumen); a longer and broader pistillate zone, 2.5–3 mm long and 4.5–5 mm in diameter at the base, comprising 4–5 rows of densely congested pistils (vs. ca. 1.5 mm long and ca. 4 mm in diameter, comprising 2–3 rows); larger ovaries, 1.5–1.7 × 0.6–0.7 mm, obovoid to oblong (vs. ca. 0.7 × 0.5 mm, obovoid); larger stigmas, 0.45–0.55 mm in diameter (vs. ca. 0.3 mm); a broader sterile interstice, 1.4–1.5 mm in diameter (vs. 0.8–1.1 mm in diameter); mutually distant staminodes with their apices directed downward to slightly downward (vs. lower staminodes congested, upper ones more widely spaced, and their apices directed upward to nearly perpendicular to the spadix axis); a longer staminate zone, 4–6 mm long, comprising 6–7 whorls of stamens (vs. 2.8–3.0 mm long, comprising 4–6 whorls); an appendix with an abruptly but slightly expanded base (vs. not abruptly expanded); and a longer stipe, 7–10 mm long (vs. ca. 5 mm). A detailed comparison of the diagnostic morphological characters of the new species and its most closely related species is provided in Table 1.

### 3.4. Description

Small, deciduous herbs, up to 35 cm tall. Tubers hypogeal, globose to subglobose, 2–3.5 cm in diameter, pale to dark brown externally, white internally. Roots filiform, 1–1.5 mm in diameter, white. Leaves 1–2, produced together. Petioles 10–15 cm long, 4–6 mm in diameter at the base, gradually narrowing toward the apex, erect, glabrous, cylindrical, and almost entirely terete, except distally, where they are shallowly canaliculate dorsally; basal subterranean portion white; upper aerial portion dark brown to dark greyish brown, grading into green toward the apex. Petiolar sheath 3.5–5 cm long, ca. one-third of the petiole length. Leaf blade mostly narrowly elliptic, rarely elliptic, 13–16 × 4–5 cm, chartaceous, medium green adaxially, pale green abaxially, glabrous on both surfaces, margin entire or undulate, base cuneate, apex shortly acute to shortly acuminate; midrib impressed adaxially, raised and rounded abaxially, 2–3 mm wide at the base, gradually narrowing toward the apex of the blade; primary lateral veins 7–9 on each side, impressed adaxially and raised abaxially, diverging from the midrib and anastomosing 0.5–4.5 mm from the margin to form a submarginal (intramarginal) vein; interprimary veins slightly less conspicuous than the primary lateral veins; higher-order venation reticulate. Inflorescences 1–3 together, emerging simultaneously with the leaves, each subtended by a cataphyll. Cataphyll 2.5–3 cm long, lanceolate to triangular-ovate, membranous, white, becoming brown upon withering. Peduncle 1.5–2 cm long, 2–4 mm in diameter, almost entirely subterranean, terete, glabrous, white. Spathe 7–9 cm long, distinctly differentiated by a constriction into a spathe base and a spathe limb. Spathe base ovoid, 14–16 mm long, 10–11 mm in diameter, glabrous, pale purple to pale purplish blue externally, with pale to dark purple spots or mottling, paler and lacking spots or mottling internally, margins free and convolute; spathe limb narrowly elongate-ovate, 5.5–6.5 cm long, 0.8–1 cm wide at its widest point near the base, concolorous with the spathe base; apex narrowly acute, blunt, or obtuse. Spathe limb erect and gaping at early pistillate anthesis, exposing the staminate zone, then strongly reflexed during pistillate anthesis; at late pistillate anthesis, it gradually becomes erect, subsequently curves forward, and closes over the staminate zone by convolution of the lower margins, leaving a partial opening for the appendix; the upper margins convolute except distally; the limb strongly forward-curved during staminate and post-staminate anthesis. Spadix sessile, 5–5.5 cm long, shorter than the spathe; pistillate zone 2.5–3 mm long, 4.5–5 mm in diameter at the base, shortly conical, comprising 4–5 rows of densely congested pistils; ovary 1.5–1.7 mm high, 0.6–0.7 mm in diameter, obovoid to oblong, white, unilocular, with a single basal ovule borne obliquely on a funicle arising from the basal placenta; stigma sessile, discoid, 0.45–0.55 mm in diameter, dark red, papillate; sterile interstice between the pistillate and staminate zones 11–12 mm long, 1.4–1.5 mm in diameter; upper portion naked, terete, glabrous, and white; lower portion covered with 4–5 spiral rows of staminodes; staminodes subulate, 1.5–3.2 mm long, 0.55–0.75 mm in diameter, oriented nearly perpendicular to the spadix axis or slightly curved upward, glabrous, yellow, mutually distant; apex obtuse, pointing downward to slightly downward; staminate zone 4–6 mm long, 2.4–2.6 mm in diameter, shortly cylindrical, comprising 6–7 whorls of stamens; stamens congested, solitary, not grouped into multistaminate flowers, 0.6–0.7 mm in diameter, orange; appendix stipitate, 2.8–3.2 cm long excluding the stipe, 2–2.5 mm in diameter at the base, subcylindrical, tapering toward the apex, erect, glabrous, reddish brown; apex acute; base abruptly but only slightly expanded; stipe cylindrical, 7–10 mm long, 1–1.7 mm in diameter, glabrous, white. Infructescence ovoid 1.8–2 cm long, 1.4–1.6 cm in diameter, infructescence peduncle 5–6 cm long. Berries asymmetrically clavate, 6–11 mm long, 2.4–3.6 mm in diameter.

### 3.5. Phenology

The new species flowers from May to early June and produces mature fruits from late June to July.

### 3.6. Distribution and Habitat

**Figure 5:**
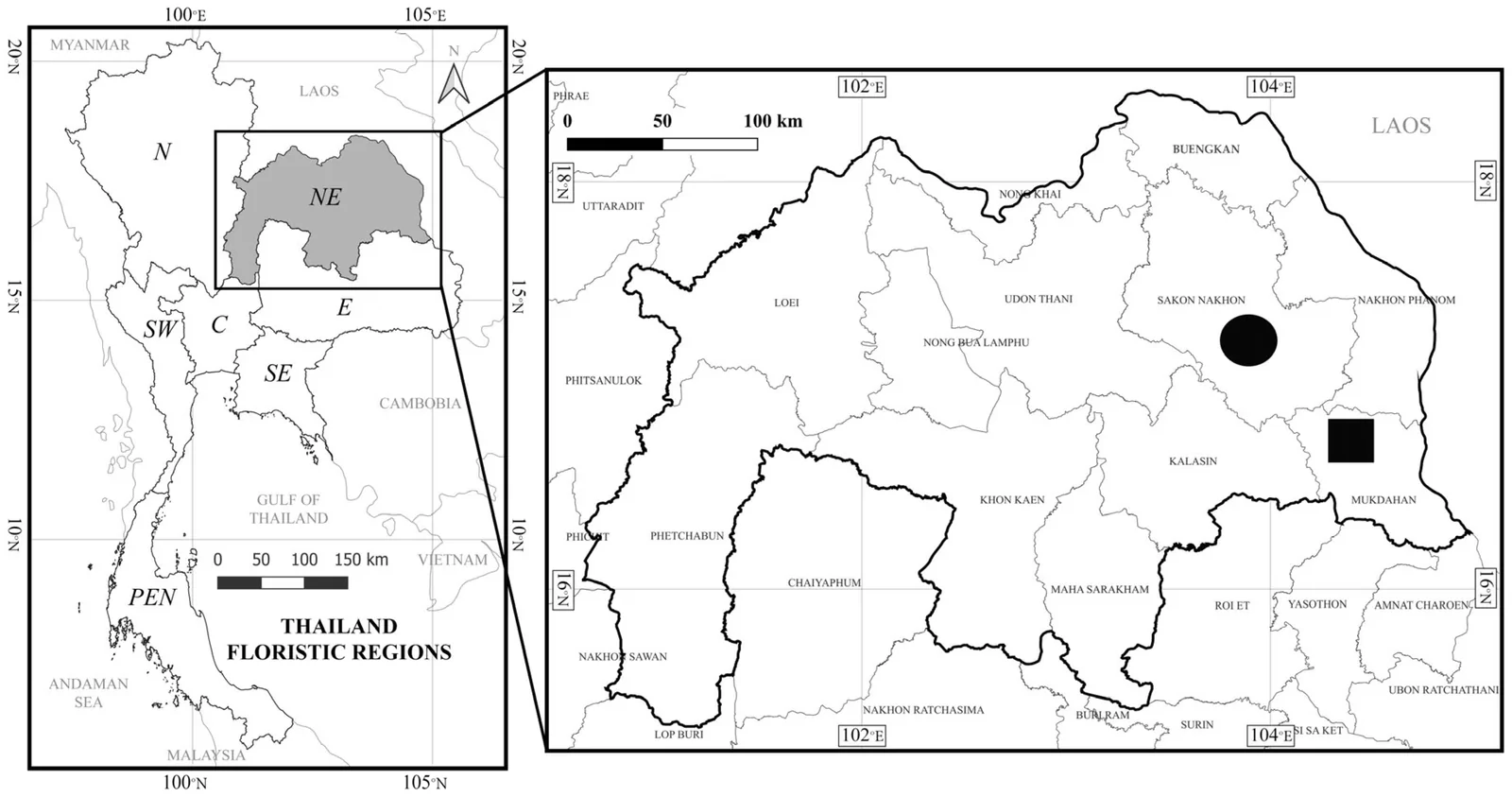
Distribution map of *Typhonium boonkerdii* in Mukdahan Province (black square) and *T. cucullatum* K.Z.Hein, P.Saensouk & Saensouk from Sakon Nakhon Province (black circle), in northeastern Thailand.

The species is presently known only from its type locality in Mukdahan Province, northeastern Thailand. It inhabits mixed deciduous forest at elevations of 300–400 m, growing in shaded sites on soil overlain by a variable layer of leaf litter, ranging from thin to thick and occurring at only a single known locality in mixed deciduous forest. The dominant plant species in this area include trees such as *Afzelia xylocarpa* (Kurz) Craib, *Dalbergia oliveri* Gamble ex Prain, *Lagerstroemia calyculata* Kurz, *Pongamia brandisiana* (Kurz) Z.Q.Song, *Pterocarpus macrocarpus* Kurz and *Xylia xylocarpa* (Roxb.) W.Theob.; shrubs and herbs such as *Bauhinia thailandica* Chatan & Promprom, Clerodendum sp and *Amorphophallus coudercii* (Bogner) Bogner and *A. sakonnakhonensis* Chatan & Promprom. Within the population, both juvenile and mature individuals were observed, occurring either in small groups or as isolated individuals. (Figure 5).

### 3.7. Etymology

The specific epithet honors Professor Emeritus Dr. Thaweesakdi Boonkerd of Chulalongkorn University, Thailand, in recognition of his contributions to plant taxonomy, particularly descriptive taxonomy and plant biosystematics in Thailand.

### 3.8. Vernacular Name

Bon Boonthawee.

### 3.9. Preliminary Conservation Assessment

A preliminary conservation assessment conducted in accordance with the IUCN Red List Categories and Criteria supports the assessment of this species as Critically Endangered (CR) under criterion D because of its extremely small population size [22]. Fewer than 50 mature individuals are estimated to occur at the known locality, and the species is currently known from only a single population. Potential threats to the species at the type locality include habitat disturbance associated with the collection of non-timber forest products by local communities. Such activities may result in the accidental trampling and damage of plants, particularly during the flowering period, potentially disrupting successful reproduction and recruitment. In addition, recurrent forest fires may expose the underground tubers to excessive heat, causing tissue damage and potentially delaying subsequent growth, reducing population recovery, and limiting the species’ local spread.

For the conservation of this species, recommended measures include restricting access to the population during the flowering period, raising awareness among local communities, establishing a regular population-monitoring programme, and implementing appropriate fire-management practices. Ex situ conservation through the collection and preservation of seeds or tubers should also be considered to reduce the risk of local extinction.

## 4. Discussion

*Typhonium boonkerdii* is morphologically most similar to *T. cucullatum*, a species described from northeastern Thailand [7]. The two species share several features, including a small, seasonally dormant tuberous habit; elliptic to narrowly elliptic leaf blades; a similar overall organization of the spathe and spadix; and a comparable pattern of spathe-limb movement during anthesis. Accordingly, *T. cucullatum* was selected as the principal species for detailed morphological comparison. The similar movement of the spathe limb, however, does not distinguish the two taxa and is therefore not regarded as a diagnostic character.

Our study indicates that the morphological distinctiveness of *T. boonkerdii* is supported primarily by a combination of vegetative and reproductive characters rather than by any single diagnostic feature. The most taxonomically informative characters are associated with the reproductive structures, particularly the number of staminate and pistillate rows, the arrangement and orientation of the staminodes, and the morphology of the appendix base and its stipe. The shape of the leaf-blade apex provides an additional vegetative distinction. In the mature individuals examined, these characters consistently occurred together as a coherent morphological pattern. Such a combination of correlated characters provides stronger taxonomic support than an isolated difference in the size of a single structure. A detailed character-by-character comparison is provided in Table 1 and is therefore not repeated here.

Quantitative differences in the dimensions of the pistillate zone, ovaries, stigmas, sterile interstice, staminate zone, and appendix stipe provide additional evidence supporting the distinction between the two taxa. Nevertheless, these measurements require cautious interpretation. The data for *T. boonkerdii* represent ranges observed in the available material, whereas those for *T. cucullatum* were obtained from its original description and available images of the type specimens rather than from population-level sampling. Consequently, inferential statistical comparisons could not be performed, and the available data do not demonstrate that any single quantitative difference independently exceeds the complete range of intraspecific variation in *T. cucullatum*. For this reason, ovary size, stigma diameter, and other dimensional characters are treated as supporting evidence and not as independently decisive characters.

The present study also lacks molecular evidence. We therefore do not claim to have demonstrated genetic divergence, reproductive isolation, or a close phylogenetic relationship between *T. boonkerdii* and *T. cucullatum*. Within the framework of morphology-based taxonomy, the consistent combination of vegetative and reproductive characters supports the recognition of *T. boonkerdii* as a morphologically well-delimited species hypothesis. This interpretation is based on comparisons of living plants and voucher specimens of the new species with the original description and available images of the type specimens of *T. cucullatum*. It should therefore be regarded as a testable taxonomic hypothesis that can be further evaluated through additional morphological, population-level, and molecular evidence.

The fact that *T. boonkerdii* is currently known from a single population represents an important limitation of the present study. Nevertheless, the study area has been surveyed repeatedly during numerous field trips, providing substantial field-based observations of the known population. Broader field surveys are still needed to determine whether the species occurs beyond the type locality and to document the full range of morphological variation within the taxon. Future surveys should, where feasible, extend to other potentially suitable habitats across Thailand and neighboring countries in order to better assess its geographical distribution, population variation, and taxonomic boundaries. Because species of *Typhonium* are seasonally dormant and may be visible above ground for only a limited period each year, field surveys conducted across the vegetative, flowering, and fruiting seasons will be particularly important. Population-level sampling of both *T. boonkerdii* and *T. cucullatum*, combined with DNA-based phylogenetic analyses, will be needed to clarify their evolutionary relationships and to further evaluate the stability of the proposed species boundary. Meanwhile, the presently known occurrence of *T. boonkerdii* in only a single, small population highlights the importance of continued population monitoring and habitat protection.

The agreement between the morphological characters in the diagnosis and the comparative data supports the taxonomic recognition of *T. boonkerdii* as a distinct species. To distinguish the new species from morphologically similar taxa, an identification key is presented below.

Key to the morphologically similar species:
1Leaf blade apices shortly acute to shortly acuminate; ovaries larger, 1.5–1.7 × 0.6–0.7 mm; staminodes with their apices directed downward to slightly downward*T. boonkerdii*1Leaf blade apices tapering into a long, slender acumen; ovary smaller, ca. 0.7 × 0.5 mm; staminode with their apices directed upward to nearly perpendicular to the spadix axis*T. cucullatum*

## Figures and Tables

**Table 1:** Comparative morphology of new species to most similar species, *Typhonium cucullatum* K.Z.Hein, P.Saensouk & Saensouk.

Charaters	*Typhonium boonkerdii*	*Typhonium cucullatum*
Tubers	2–3.5 cm in diameter, pale to dark brown externally	2.0–2.5 cm in diameter, externally pale brown
Leaves	1–2, produced together	2–3 together
Petioles	4–6 mm in diameter at the base	2.5–3.0 mm in diameter,
Petiolar sheath	3.5–5 cm long, ca. one-third of the petiole length	6.5–7.0 cm long, ca. 1/2 of petiole length
leaf blade apex	shortly acute to shortly acuminate	caudate, tapering into a long, slender acumen
Inflorescences	emerging simultaneously with the leaves	emerging either before or simultaneously with the leaves
Cataphyll	2.5–3 cm long	up to 2 cm long
Peduncle	1.5–2 cm long, 2–4 mm in diameter	ca. 1.3 cm long, 2.5–3.0 mm in diameter
Spathe base	ovoid, 14–16 mm long, 10–11 mm in diameter	ellipsoid-ovoid, ca. 1.5 cm long, 0.8–0.9 cm in diameter
Pistillate zone	2.5–3 mm long, 4.5–5 mm in diameter at the base, shortly conical, comprising 4–5 rows of densely congested pistils	ca. 1.5 mm long, ca. 4 mm in diameter at the base, shortly conical or hemispheroid, comprising 2–3 rows of congested pistils
Ovary	1.5–1.7 mm high, 0.6–0.7 mm in diameter, obovoid to oblong	ca. 0.7 mm high, ca. 0.5 mm in diameter, obovoid
Stigma	discoid, 0.45–0.55 mm in diameter, dark red	discoid, ca. 0.3 mm in diameter, red
Sterile interstice	11–12 mm long, 1.4–1.5 mm in diameter; lower portion covered with 4–5 spiral rows of staminodes	1.2–1.4 cm long, 0.8–1.1 mm in diameter, lower part covered with 5–6 spirals of staminodes
Staminodes	subulate, 0.55–0.75 mm in diameter, mutually distant; apices obtuse, pointing downward to slightly downward	subulate, ca. 0.5 mm in diameter; lower ones congested, upper ones more widely spaced; apices acute or obtuse, pointing upward to nearly perpendicular to the spadix axis in the upper ones
Staminate zone	4–6 mm long, comprising 6–7 whorls of stamens	2.8–3.0 mm long, comprising 4–6 whorls of stamens
Appendix	reddish brown, base abruptly expanded, but only slightly	pale orange, base not abruptly expanded
Stipe	7–10 mm long, 1–1.7 mm in diameter	ca. 0.5 cm long, 1.7 mm in diameter

## Data Availability

All of the data supporting the findings of this study are available in the main text.
